# Antimicrobial Activity of Ohelo Berry (*Vaccinium calycinum*) Juice against *Listeria monocytogenes* and Its Potential for Milk Preservation

**DOI:** 10.3390/microorganisms10030548

**Published:** 2022-03-02

**Authors:** Biyu Wu, Xiaohan Liu, Stuart T. Nakamoto, Marisa Wall, Yong Li

**Affiliations:** 1 Department of Human Nutrition, Food and Animal Sciences, University of Hawaii at Manoa, 1955 East West Road, Agricultural Sciences Building 216, Honolulu, HI 96822, USA; biyu@hawaii.edu (B.W.); felxh6005@mail.scut.edu.cn (X.L.); snakamo@hawaii.edu (S.T.N.); 2 Daniel K. Inouye U.S. Pacific Basin Agricultural Research Center, 64 Nowelo Street, Hilo, HI 96720, USA; marisa.wall@usda.gov

**Keywords:** ohelo berry, polyphenols, antimicrobial effect, *Listeria monocytogenes*, physicochemical properties, biofilm formation, gene expression, milk preservation

## Abstract

*Listeria monocytogenes* is a foodborne pathogen and causes illnesses with a high mortality rate in susceptible populations. Several dairy-related outbreaks have been attributed to contamination by *L. monocytogenes*, which requires antimicrobial interventions to enhance the safety of these products. This study aimed to determine the antimicrobial activity of the ohelo berry (*Vaccinium calycinum*), a Hawaiian wild relative of cranberry, against *L. monocytogenes* in culture media and milk products. The effect of ohelo berry juice at its sub-inhibitory concentrations on the physicochemical properties, biofilm formation, and gene expression of *L. monocytogenes* was also investigated. The minimum inhibitory concentration of ohelo berry juice against *L. monocytogenes* was 12.5%. The sub-inhibitory concentration of ohelo berry juice (6.25%) significantly increased the auto-aggregation and decreased the hydrophobicity, swimming motility, swarming motility, and biofilm formation capability of *L. monocytogenes*. The relative expression of genes for motility (*flaA*), biofilm formation and disinfectant resistance (*sigB*), invasion (*iap*), listeriolysin (*hly*), and phospholipase (*plcA*) was significantly downregulated in *L. monocytogenes* treated by the 6.25% juice. *L. monocytogenes* was significantly inhibited in whole and skim milk supplemented with 50% ohelo berry juice, regardless of the fat content. These findings highlight the potential of ohelo berry as a natural preservative and functional food to prevent *L. monocytogene*s infection.

## 1. Introduction

Foodborne illness outbreaks not only pose a serious threat to public health but also lead to substantial economic losses for society. *Listeria monocytogenes* is a dangerous foodborne pathogen and can cause a severe disease named listeriosis, with a high mortality rate in susceptible populations. The Centers for Disease Control and Prevention (CDC) estimates that 1600 people have listeriosis, with around 260 deaths annually in the United States [1]. *L. monocytogenes* is a rod-shaped, gram-positive bacterium. It is capable of adapting to and surviving under adverse conditions, such as low temperature, low pH, and high salt concentrations [2]. *L. monocytogenes* is ubiquitously present in the environment and can be found in soil, wastewater, vegetation, wild animals, livestock, etc. [3].

It was recently reported that more than 75% of *L. monocytogenes* outbreaks were linked to contaminated dairy products and fruits between 2015 and 2019 [4]. Moreover, the consumption of soft cheese made from unpasteurized milk or under unsanitary conditions was recognized as a potentially serious risk, especially to pregnant women and persons with weak immune systems [4]. Control of *L. monocytogenes* in food production remains a great challenge. Therefore, effective antimicrobial approaches for preventing food contamination or the occurrence of listeriosis are urgently needed.

Nowadays, consumers tend to choose more natural, healthy, and safe food [5] because inappropriate use of antimicrobial agents in food production might result in undesirable residues in food and the emergence of antimicrobial-resistant microorganisms [6]. Moreover, consuming synthetic food preservatives may cause health concerns, including potential side effects and increased cancer risks [7]. Thus, the use of natural antimicrobials in food preservation has attracted increasing attention from scientists, food manufacturers, and consumers [6]. 

Phytochemicals, especially polyphenolic compounds, have bioactive functions. Studies have also demonstrated that polyphenolic compounds in berries hold antimicrobial properties against various pathogens [8,9,10,11]. Additionally, the phytochemicals extracted from fruits have been proven to exhibit multiple effects on human health. The antioxidant activity of polyphenols provides them with functions such as anti-inflammation, anti-tumor, prevention of cardiovascular diseases, and delaying aging [12,13]. 

Cranberry (*Vaccinium macrocarpon*) was found to have the highest phenolic contents among the 20 common fruits in America [14] and exhibited strong antimicrobial activities against common foodborne pathogenic bacteria [11,12,15]. Ohelo berry is a wild relative of cranberry and grows in Hawaii only. It typically grows at elevations between 640 and 3700 m on disturbed volcanic sites on the islands of Maui and Hawaiʻi [16]. Residents usually gather ripened ohelo berries to make jam, jelly, and other foods between August and September [17]. The two most common species of ohelo berry are low-growing *Vaccinium reticulatum* and highbush *Vaccinium calycinum* [17]. *V*. *calycinum* contains a higher level of phytochemicals than *V*. *reticulatum*, and the total phenolic content of *V*. *calycinum* is seven times that of cranberry [18].

To date, there have been no reports on the antimicrobial properties of ohelo berry. This study aimed to measure the phytochemical contents of ohelo berry juice and determine its antimicrobial activities. Physicochemical properties and biofilm formation capability of *L. monocytogenes* treated with sublethal concentrations of ohelo berry juice were evaluated. Efforts were also made to investigate the influence of ohelo berry juice on the gene expression of *L. monocytogenes*. The potential of ohelo berry juice as a food preservative was explored in whole milk and skim milk with *L. monocytogenes*.

## 2. Materials and Methods

### 2.1. Ohelo Berry Juice Preparation

Ohelo berries (*V. calycinum*) were harvested from Hawaiʻi Island and stored at −80 °C. For the following analysis, thawed ohelo berries were squeezed and filtered to obtain juice. The juice was filter-sterilized using a sterile syringe filter with 0.2 μm polyethersulfone membrane (VWR) before all experiments of analyzing its antimicrobial activities were performed. 

### 2.2. Chemical Analysis of Ohelo Berry Juice

#### 2.2.1. Quantification of Sugar and Organic Acids in Ohelo Berry Juice

Ohelo berry juice was analyzed for the ratio of °Bx to titratable organic acids. The pH of the sample was measured using a pH meter (Oaklon). A refractometer (Bio-Rad, iMark) was used to quantify soluble sugar solids. The concentration of organic acids in citric acid equivalents was determined by titration with 0.1 M NaOH [19]. 

#### 2.2.2. Total Phenolic Concentration

The total phenolics in ohelo berry juice were measured by the Folin Ciocalteu method [20]. Gallic acid stock solution was prepared by dissolving 0.5 g of gallic acid monohydrate (Sigma, St. Louis, MO, USA) in 10 mL of ethanol and then diluting to 100 mL with distilled water (5 mg/mL). Various concentrations of gallic acid solutions (25, 50, 100, 250, and 500 μg/mL) were prepared from stock as gallic acid calibration standards. A volume of 30 µL of distilled water (control), gallic acid calibration standards, and diluted ohelo berry juice were pipetted separately into wells of a 96-well plate (Costar, Somerville, MA, USA). Then, 210 µL of water, 15 µL of Folin Ciocalteu reagent (EMD Millipore Corporation, Merck KGaA, Darmstadt, Germany), and 45 µL of sodium carbonate solution were added sequentially into each well. The plate was incubated in the dark at room temperature for 2 h. The absorbance was measured at 765 nm using a spectrophotometer (Bio-Rad, iMark, Hercules, CA, USA). After that, a standard curve was created from the readings of gallic acid standards. All the experiments were carried out in triplicates, and the average absorbance values obtained at different concentrations of gallic acid solution were used to plot the calibration curve. The concentration of phenolic compounds in ohelo berry juice was calculated using the standard curve and reported in gallic acid equivalents (GAE).

#### 2.2.3. Anthocyanin Concentration

The concentration of anthocyanins in ohelo berry juice was determined using the pH differential method and reported in equivalents of cyanidin-3-glucoside [21]. Two buffers were prepared for this test. The pH 1.0 buffer is 0.025 M of potassium chloride, and the pH 4.5 buffer is 0.4 M of sodium acetate. The sample was diluted with the two buffers separately. Ohelo berry juice was diluted with pH 1.0 buffer until the absorbance at 520 nm was within the linear range of the spectrophotometer. Furthermore, the samples were diluted at least 5-fold with the buffers so that the buffering capacity of the reagents was not exceeded. The same dilution for pH 1.0 buffer was used for pH 4.5 buffer and followed by measuring absorbance at both 520 and 700 nm within 30 min of the samples being prepared, with a blank well filled with distilled water as control. The anthocyanin content was calculated according to the equation described by Lee et al. [21].

### 2.3. Bacterial Strain and Growth Conditions

*L. monocytogenes* F2365 was used for analyzing the antimicrobial activities of ohelo berry juice. The strain was stored at −80 °C as frozen stock culture. *L. monocytogenes* was streaked on modified Oxford agar (MOX, BD, Franklin Lakes, NJ, USA) and then grown in tryptic soy broth (TSB, BD, Franklin Lakes, NJ, USA) at 37 °C for 24 h before being used in the following tests.

### 2.4. Determination of the Minimum Inhibitory Concentration (MIC) and Minimum Bactericidal Concentrations (MBC) of Ohelo Berry Juice

The antimicrobial efficacy of ohelo berry juice was determined by the standard methods of Clinical and Laboratory Standards Institute by evaluating the growth of microorganisms in microbiological broth [22]. The *L. monocytogenes* culture was 10-fold serially diluted to approximately 6 log CFU/mL with 2× Mueller–Hinton (MH) broth. Ohelo berry juice was two-fold serially diluted with sterilized water to obtain 0-fold, 2-fold, 4-fold, 8-fold, 16-fold, 32-fold, and 64-fold dilutions. Then, the same volumes of 2× MH broth with 6 log CFU/mL *L. monocytogenes* and serially diluted ohelo berry juice were mixed to yield 50%, 25%, 12.5%, 6.25%, 3.125%, 1.56%, or 0.78% of ohelo berry juice, and 5 log CFU/mL of bacteria. A positive control was established to be 1:1 of sterilized water and 2× MH broth with 6 log CFU/mL of bacteria. A negative control was established by mixing sterilized water with un-inoculated 2× MH broth. The cocktails were incubated at 37 °C for 24 h. Viable cell counts were determined at 0 and 24 h on plate count agar (PCA). Plates were incubated at 37 °C for 24 h before enumeration. Minimum inhibitory concentration (MIC) was determined as the lowest concentration of the juice that inhibited the growth of *L. monocytogenes* after incubation. Minimum bactericidal concentration (MBC) was determined as the lowest concentration of the juice that killed *L. monocytogenes* after incubation [23]. MIC and MBC were determined by comparing viable cell counts between the control and treatments at 0 and 24 h. The procedures were modified from the methodology described by Lacombe et al. [24], where MH broth was used in the present study. 

### 2.5. Growth Inhibition Kinetics of Ohelo Berry Juice against L. monocytogenes

The *L. monocytogenes* culture was 10-fold serially diluted to approximately 5 log CFU/mL with 2× MH broth. The same volume of serially diluted ohelo berry juice was added to the medium to achieve final concentrations of 50%, 25%, 12.5%, and 6.25%. The samples were incubated at 37 °C for 24 h and collected at 0, 2, 4, 8, and 24 h for plating on PCA. The plates were incubated at 37 °C for 24 h before enumeration. The growth inhibition kinetic curve was prepared by plotting bacterial concentration versus time. 

### 2.6. Evaluation of the Physicochemical Properties and Biofilm Formation Capability of L. monocytogenes Treated with Ohelo Berry Juice

#### 2.6.1. Hydrophobicity

Hydrophobicity was determined according to the method described by Salaheen et al. [25]. *L. monocytogenes* was grown in Luria–Bertani (LB) broth in the absence and presence of predetermined sub-inhibitory concentrations (½ MIC and ¼ MIC) of ohelo berry juice at 37 °C for 24 h. Each culture was centrifuged at 3000 g for 20 min. The cells were thoroughly suspended in 5 mL of phosphate buffer saline (PBS, pH 7.2). The absorption of suspension was measured at 570 nm using a spectrophotometer (UV-1600PC, VWR) and adjusted to approximately 0.5 (Ht0) with PBS. After that, 2 mL of each adjusted solution was transferred to a new tube and mixed with 1 mL of n-hexadecane, followed by incubation at room temperature for 5 min. The hexadecane phase was removed, and the absorption of the aqueous phase was measured at 570 nm (Ht5). Hydrophobicity was calculated using the equation as follows:

Hydrophobicity (%) = (1 − Ht5/Ht0) × 100(1)

#### 2.6.2. Auto-Aggregation

*L. monocytogenes* cells were treated with sub-inhibitory concentrations of ohelo berry juice as described above. After the cells were collected, they were thoroughly suspended in 5 mL of PBS. The absorption of cell suspension was measured at 570 nm and adjusted to approximately 0.5 (At0) with PBS. A volume of 3 mL of adjusted cell solution was transferred to a new tube and incubated at 37 °C for 2 h. After that, the absorption of the supernatant was measured at 570 nm (At5). Auto-aggregation was calculated by the equation below [25]:

Auto-aggregation (%) = (1 − At5/At0) × 100(2)

#### 2.6.3. Motility

Motility was determined via an assay described previously, with minor modifications [26]. Firstly, 0.2% and 0.5% LB agar were prepared for swimming and swarming motility tests, respectively. The lower agar density for swimming motility in the present study allowed bacteria to move within the agar easily. Two milliliters of 62.5% and 31.25% ohelo berry juice were mixed with 18 mL warm LB agar at around 50 °C and poured into Petri dishes, representing 6.25% (½ MIC) and 3.125% (¼ MIC) of ohelo berry juice in the agar, respectively. An equal amount of water was used as a negative control. After the agar was solidified, 2 µL of fresh *L. monocytogenes* culture was spotted in the center of the agar. The plates were incubated at 37 °C for 24 to 48 h. The migration of bacteria from the point of inoculation after incubation reflected their motility. The diameter of the growth zone was measured for control and the treatments.

#### 2.6.4. Biofilm Formation

The procedures for this assay were modified from a method described previously [27]. Fresh *L. monocytogenes* culture was 10-fold diluted 4 times with 2× MH broth. Aliquots (100 μL) of diluted *L. monocytogenes* culture were transferred into wells of a sterile polystyrene, flat-bottom, 96-well plate. Then, 100 μL of MIC and ½ MIC ohelo berry juice were mixed in triplicate with bacterial cultures to a final concentration of ½ MIC and ¼ MIC, respectively. Further, un-inoculated MH broth with the ohelo juice dilutions (100 μL) was prepared in triplicate to serve as negative controls. The plates were incubated at 37 °C for 48 h, followed by decanting the broth from each well and rinsing gently three times with PBS. In the present study, the plate was allowed to dry at 75 °C for 30 min. Then 210 μL of 0.4% (wt/vol) crystal violet (CV) was added to each well, and the plate was incubated at room temperature for 15 min. Unabsorbed CV in each well was removed, and the well was rinsed with deionized water. Subsequently, 210 μL of absolute ethanol was loaded into the well to solubilize the CV adsorbed to the biomass, and the plate was incubated at room temperature for 10 min. The absorbance was measured at 570 nm using a microplate reader (Biotek Synergy LX, BioTek Instruments, Santa Clara, CA, USA) to quantify the solubilized CV. Negative controls with cell-free broth were used to adjust for background signals. 

### 2.7. RT-qPCR

Quantitative reverse transcription-polymerase chain reaction (RT-qPCR) was employed to quantify the expression of selected genes in *L. monocytogenes* treated with ohelo berry juice, using a method described by Wu et al. [28]. *L. monocytogenes* was grown in MH broth in the absence and presence of ½ MIC and ¼ MIC of ohelo berry juice at 37 °C for 24 h. Then, 450 μL of each culture was transferred to a 1.7 mL microcentrifuge tube for RNA extraction using RNeasy Mini Kit (Qiagen, Hilden, Germany), following the manufacturer’s instructions. Concentrations of RNA extracts were measured using Nanophotometer P-Class 300 (Implen, Munich, Germany) and adjusted for further purification with RQ1 DNase (Promega, Madison, WI, USA) to remove DNA contamination. The concentrations of digested RNA were measured again and reverse-transcribed using the High-Capacity cDNA Reverse Transcription Kit (Applied Biosystems, Waltham, MA, USA). Briefly, 5 μg of digested RNA was mixed with 10 μL of 2× RT master mix and nuclease-free H_2_O to a total volume of 20 μL. The synthesis reaction was performed in the MJ Mini Thermal Cycler (Bio-Rad, Hercules, CA, USA) at 25 °C for 10 min, 37 °C for 120 min, and 85 °C for 5 min. The cDNA samples were stored at −20 °C. Quantitative PCR was performed using GoTaq qPCR Master Mix (Promega) as follows: 1 μL cDNA, 1 μL of forward primer (50 µM), 1 μL of reverse primer (50 μM), 10 μL of 2× GoTaq qPCR Master Mix, and 7 μL of Nuclease-free H_2_O. The amplification was performed in CFX96 Real-Time Detection Systems (Bio-Rad), and the thermal cycling parameters were set with an initial denaturation at 95 °C for 2 min, followed by 40 cycles of denaturation at 95 °C for 15 s, annealing at 60 °C for 1 min, and extension at 72 °C for 1 min. Information about the primers used in quantitative PCR is shown in Table 1. The relative expression levels of genes were calculated by the comparative method using CFX Maestro^TM^ Software (Bio-Rad) [29]. The housekeeping gene, 16S *rRNA*, was used as the reference gene for the normalization of the target gene expression [30].

### 2.8. Effect of Ohelo Berry Juice on the Growth of L. monocytogenes in Milk

The effectiveness of ohelo berry juice on suppressing the growth of *L. monocytogenes* in milk was investigated. Ultra-high-temperature whole milk and skim milk were purchased from a local supermarket. The *L. monocytogenes* culture was 10-fold serially diluted with peptone water (0.1%), and 0.1 mL of bacterial dilutions was added into 10 mL of milk containing 12.5% (MIC), 25% (2 MIC), and 50% (MBC) ohelo berry juice to achieve a bacterial concentration of 6 log CFU/mL. Inoculated milk (10 mL) without ohelo berry juice was included as a positive control. The milk samples were incubated at 7 or 37 °C for 72 h. Aliquots were removed at 0, 6, 24, 48, and 72 h for the bacterial count. An agar overlay method was employed to recover injured bacterial cells [32]. Briefly,10-fold serial dilutions of the samples were spread on PCA. The plates were incubated at 37 °C for 2 h for the resuscitation of injured cells. After the 2-h preincubation, 10 mL of melted modified Oxford Agar supplemented with Moxalactam Supplement (Sigma-Aldrich) were poured onto PCA, allowed to solidify, and incubated at 37 °C for 48 h. Colonies of a brown color surrounded by a black zone were recorded as *L. monocytogenes*. 

### 2.9. Statistical Analysis

All experiments were performed in three independent replicates. The viable cell counts of *L. monocytogenes* were converted to log CFU/mL, and means and standard deviations were calculated by using Microsoft Excel (Version 2201). The physicochemical properties, biofilm formation capability, and gene expression patterns of *L. monocytogenes* treated with different concentrations of ohelo berry juice were compared. The data were analyzed by analysis of variance (ANOVA) and Tukey’s multiple comparison test to determine significant differences between treatments and storage time at *p* < 0.05 using Statistical Packages Multcomp [33] and Agricolae [34] in RStudio version 3.6.2 (Rstudio, Boston, MA, USA). 

## 3. Results

### 3.1. Chemical Analysis of Ohelo Berry Juice

Aside from acidity, phenolic compounds (especially anthocyanins) may play a considerable role in the antibacterial activity of cranberry [11,12,15]. Thus, the pH value, sugar and organic acids, total phenolics, and anthocyanin contents of ohelo berry juice were measured. Ohelo berry juice had quite a low pH of 3.27 (Table 2). The sugar and titratable acidity in ohelo berry juice was measured at a ratio of 7.6/1.42 Bx/acid. Total phenolics and anthocyanins were analyzed by the Folin Ciocalteu method and the pH differential method, respectively. The results showed the concentrations of total phenolics and anthocyanins in ohelo berry juice were 4.2 mg/mL GAE and 55.11 mg/L cyd-3-glu, respectively. 

### 3.2. Antimicrobial Effect of Ohelo Berry Juice on L. monocytogenes 

**Figure 1:**
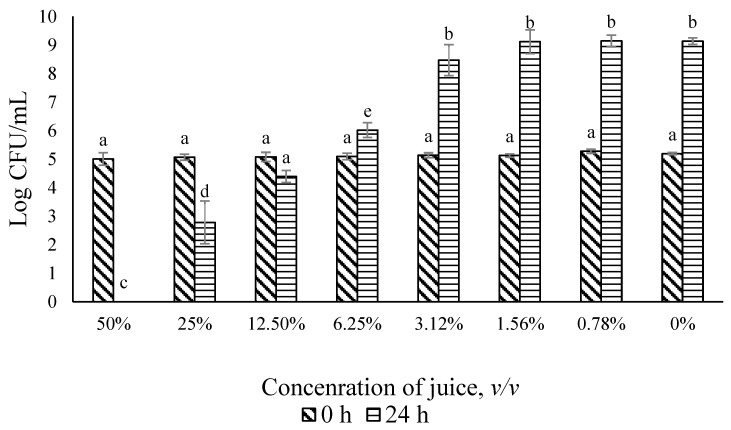
Evaluation of the antimicrobial effect of ohelo berry juice on *Listeria monocytogenes*. The viable cell counts of diluted ohelo berry juice in Mueller–Hinton broth were determined at 0 and 24 h. Means with different letters (a through e) are significantly different at *p* < 0.05.

**Figure 2:**
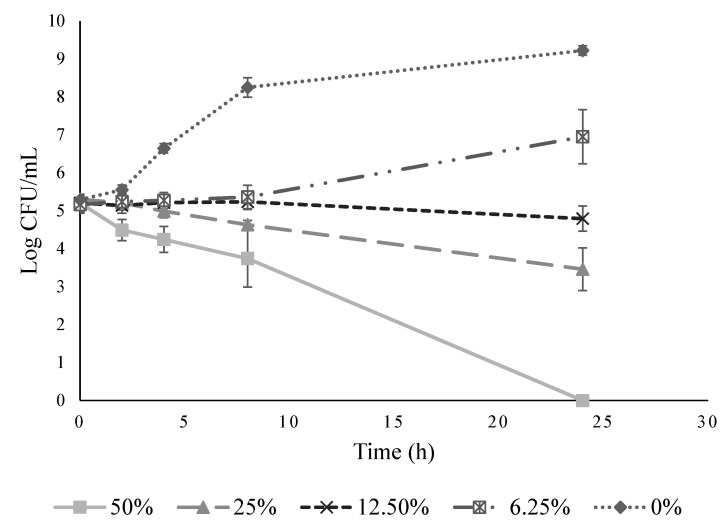
Growth inhibition kinetics of diluted ohelo berry juice (50%, 25%, 12.5%, and 6.25%) and control (0%) against *Listeria monocytogenes* in Mueller–Hinton broth.

Ohelo berry juice was added into MH broth to determine its MIC and MBC against *L*. *monocytogenes*. With dilution, the pH of ohelo berry juice gradually increased from 3.86 at 50% to 7.09 at 0.78% (Table 2). Meanwhile, the antimicrobial activity of ohelo berry juice became weaker. As shown in Figure 1, each dilution of the juice was assessed for viable cell count at 24 h, compared with the initial level at 0 h and the cell count of the control at 24 h. No significant difference was observed in the bacterial growth after 24-h incubation when 12.5% ohelo berry juice was added. Moreover, *L*. *monocytogenes* was non-detectable after 24-h incubation with 50% ohelo berry juice. Therefore, the MIC and MBC of ohelo berry juice against *L*. *monocytogenes* were 12.5% and 50%, respectively. Based on these results, the growth inhibition kinetics of the juice at concentrations of 50%, 25%, 12.5%, and 6.25% were assessed against *L*. *monocytogenes*. Figure 2 illustrates the behavior of the bacteria in diluted ohelo berry juice treatments within 24 h. Ohelo berry juice slowly killed the bacteria at an initial concentration of about 5.2 log CFU/mL. The level of *L*. *monocytogenes* decreased by 1.46 log in the 50% juice treatment from 0 to 8 h, and the bacteria was completely inactivated by 24 h. When *L*. *monocytogenes* was exposed to the MIC of ohelo berry juice in MH broth, the number of bacteria almost remained stable for the entire 24 h.

### 3.3. Effect of Ohelo Berry Juice on the Physicochemical Properties and Biofilm Formation Capability of L. monocytogenes

Ohelo berry juice showed a significant effect on tested physicochemical properties and biofilm formation capability of *L*. *monocytogenes* in microbiological media (Table 3). After being exposed to sub-inhibitory concentrations of the juice in LB broth for 24 h, *L*. *monocytogenes* decreased significantly (*p* < 0.05) in its cell surface hydrophobicity, which was 2.77% and 2.14% in the presence of 3.12% (¼ MIC) and 6.25% (½ MIC) ohelo berry juice, respectively, compared to 7.71% in the control (Table 3). In contrast, the auto-aggregation capability of *L*. *monocytogenes* cells significantly increased from 16.02% in the control to 67.98% in the 6.25% ohelo berry juice treatment (Table 3). In soft LB agar without ohelo berry juice, *L*. *monocytogenes* formed large migration zones, reflecting its strong motility. However, cells were only able to form colonies in the center of agar plates with the juice. The bacterial swimming motility decreased significantly from 100% in the control to 41.29% and 4.39% with 3.12% and 6.25% ohelo berry juice, respectively. Similarly, both juice treatments resulted in significantly lower swarming motility of *L*. *monocytogenes* than the control. Finally, ohelo berry juice helped prevent biofilm formation of *L*. *monocytogenes* on polystyrene plates. Biofilm formation capability of *L*. *monocytogenes* in MH broth decreased significantly by 45.67% and 77%m with 3.12% and 6.25% ohelo berry juice, respectively (Table 3). 

### 3.4. Effect of Ohelo Berry Juice on Gene Expression of L. monocytogenes 

**Figure 3:**
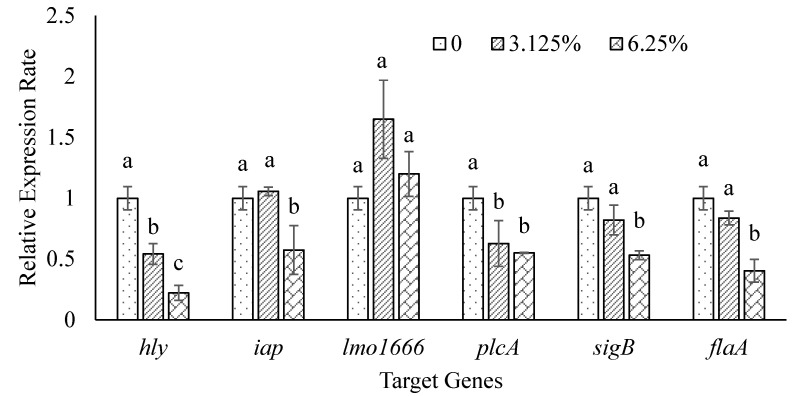
Relative expression levels of selected genes in *L. monocytogenes* after treatment with 6.25%, 3.12%, or no ohelo berry juice. Bars labeled with different letters indicate significant differences between the means of treatments at *p* < 0.05.

Quantitative RT-PCR was performed to determine the effect of ohelo berry juice on the transcription of six genes in *L. monocytogenes*, with the 16S *rRNA* gene as an internal reference gene. The quantitative PCR efficiency for the 16S *rRNA*, *hly*, *iap*, *lmo*1666, *plc*A, *sig*B, and *fla*A gene was within an acceptable range of 1.96 to 2.06 (Table 1). Relative expression of these genes was corrected for efficiency. Compared with the control, 6.25% ohelo berry juice significantly down-regulated all target genes but *lmo*1666. A significant reduction in transcript abundance was also observed for *hly* and *plc*A in *L. monocytogenes* treated with the 3.12% juice. In contrast, neither 6.25% nor 3.12% ohelo berry juice showed any significant effect on the expression of *lmo*1666 (Figure 3).

### 3.5. Effect of Ohelo Berry Juice on the Growth of L. monocytogenes in Milk

**Figure 4:**
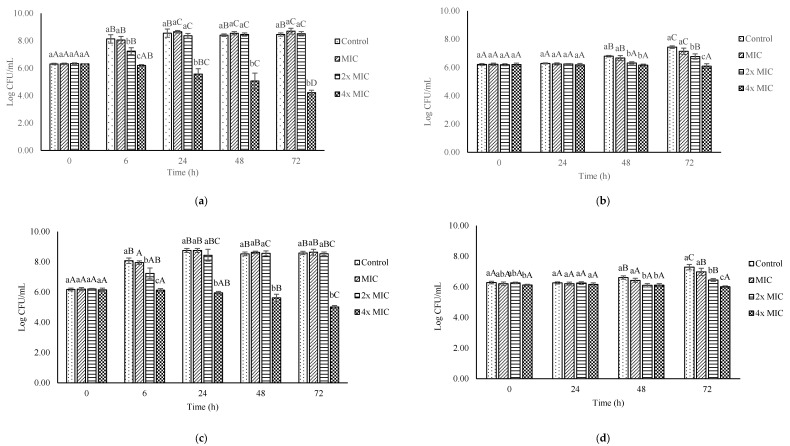
Effect of ohelo berry juice at concentrations of 12.5% (MIC), 25% (2× MIC), and 50% (4× MIC) on the growth of *L. monocytogenes* in whole milk at 37 °C (**a**), whole milk at 7 °C (**b**), skim milk at 37 °C (**c**), and skim milk at 7 °C (**d**) for 72 h. Within each time point, means with different lowercase letters are significantly different (*p* < 0.05) between the treatments. Within each treatment, means with different capital letters are significantly different (*p* < 0.05) between the time points.

The antimicrobial properties of ohelo berry juice were evaluated in whole milk and skim milk stored at 7 and 37 °C for 72 h (Figure 4). *L. monocytogenes* populations in whole milk stored at 37 °C increased from an initial level of 6.31 ± 0.05 log to 8.14 ± 0.31 log CFU/mL after 6 h and remained at around 8.5 log CFU/mL through the 72-h incubation (Figure 4A). The addition of ohelo berry juice to whole milk at 12.5% (MIC) showed no significant difference from control. When the concentration of juice increased to 25% (2× MIC), a significant inhibitory effect on *L. monocytogenes* was observed at 6 h, with the bacterial count being 7.25 ± 0.25 log CFU/mL. However, *L. monocytogenes* overcame the inhibitory effect and reached a level (8.37 ± 0.17 log CFU/mL) similar to the control at 24 h. In contrast, treatment with 50% (MBC) ohelo berry juice exhibited antimicrobial activities in a time-dependent manner and significantly reduced the number of *L. monocytogenes* in whole milk to 4.21 ± 0.18 log CFU/mL at 72 h (Figure 4A). 

Consistently, the addition of 50% ohelo berry juice to whole milk stored at 7 °C resulted in the significant inhibition of *L. monocytogenes* through a storage period of 72 h with mean counts similar to the initial level (6.10 ± 0.17 log CFU/mL) (Figure 4B). The count of *L. monocytogenes* in whole milk without juice was 6.22 ± 0.06 log CFU/mL at 0 h, then slowly reached 7.44 ± 0.09 log CFU/mL at 72 h. The 12.5% juice in whole milk at 7 °C did not limit the growth of bacteria when compared to control at any time point during incubation, whereas 25% juice in whole milk resulted in a significantly lower bacterial population compared with the control at 48 h and 72 h (Figure 4B). 

The growth of *L. monocytogenes* in skim milk incubated at 37 °C was similar to the observation in whole milk (Figure 4C). In the absence of ohelo berry juice, the count of *L. monocytogenes* increased from 6.18 ± 0.09 log to 8.08 ± 0.18 log CFU/mL at 6 h and remained at approximately 8.6 log CFU/mL. Only the treatment with 50% juice exhibited significant inhibition of *L. monocytogenes* over time, with a mean count of 5.02 ± 0.09 log CFU/mL at 72 h. Neither 12.5% nor 25% treatment showed an antimicrobial effect on *L. monocytogenes* in skim milk at 37 °C (Figure 4C).

Similar to those of whole milk treatments (Figure 4B), bacterial counts of skim milk with 12.5% ohelo berry juice incubated at 7 °C did not differ from control through 72-h storage (Figure 4D). Nevertheless, counts of skim milk with 25% juice were significantly lower than control at 48 and 72 h. When the concentration of ohelo berry juice increased to 50%, the antimicrobial effect was significantly stronger than all other treatments at 72 h (Figure 4D). The results suggest that ohelo berry juice could effectively inhibit the growth of *L. monocytogenes* in milk regardless of the fat content and could potentially be used as an antimicrobial agent as well as a natural flavoring in dairy beverages.

## 4. Discussion

In the dairy industry, it is critical to minimize the risk of *L. monocytogenes*, which is mainly associated with unpasteurized or minimally processed milk and post-pasteurization contamination from plant environments [35]. Antimicrobial interventions using natural food preservatives have garnered increasing attention in order to enhance the safety of these products. 

Cranberry is rich in antioxidants and polyphenolics that have strong antimicrobial activities and other health benefits [8,15,36]. As a Hawaiian wild relative of cranberry, ohelo berry has comparable or even higher phenolic contents than cranberry [18]. In this study, the total phenolics and anthocyanins in ohelo berry juice were 4.2 mg/mL GAE and 55.11 mg/L cyd-3-glu, respectively. According to previous reports, the total phenolic content of cranberry juice varied from 0.5 to 2 mg/mL GAE [8,37,38]. The anthocyanins in cranberry juice were in a range of 0.9 to 1.4 mg/100 mL cyd-3-glu and depends on quantification methods [21,39]. Ohelo berry juice has a pH of 3.27, higher than that of cranberry juice (2.70) [18]. The sugar and titratable acidity ratio of ohelo berry juice was 7.6/1.42 Bx/acid, which was comparable to the results of the phytochemical composition analysis of ohelo berry juice previously [18]. It is also noteworthy that cranberry and ohelo berry have different phenolic compositions and anthocyanin profiles [18], implying their distinct antimicrobial potential.

The MIC and MBC of ohelo berry juice against *L*. *monocytogenes* were determined to be 12.5% and 50%, respectively (Figure 1). The pH of 12.5% ohelo berry juice was 4.69 (Table 2), higher than the minimum growth pH (4.4) of *L*. *monocytogenes* [31]. This suggests that the antimicrobial property of ohelo berry juice may be attributed to both organic acids and other bioactive compounds. Malic, citric, shikimic, and quinic acids are present in ohelo berry [18]. Being protonated, organic acids can cross the cell membrane into bacterial cytoplasm. Adenosine 5′-triphosphate (ATP) is required to pump the protons released from the acids out of the cell. Therefore, the lack of ATP may lead to the inhibition of bacterial growth [36]. 

Since Côté et al. reported that neutralized cranberry juice still maintained its antimicrobial effect on seven bacterial strains [8], the phenolics in ohelo berry, similar to those in cranberry, may act against bacteria through various modes of action, including protein precipitation, enzyme inactivation, membrane disruption, and leakage of cellular contents [13]. Specifically, anthocyanins, a type of polyphenolic flavonoid, can inhibit bacteria by damaging cell membranes and affecting enzymatic activities [13]. In addition, the tricarboxylic acid (TCA) cycle and the biosynthesis of bacterial cells may be affected by anthocyanins [13]. *L*. *monocytogenes* is a gram-positive bacteria and has thick peptidoglycan cell walls, which are prone to damage by polyphenols [40]. Moreover, polyphenols may sequester free ions, which are essential for the survival and virulence of bacteria [41]. Thus, the antimicrobial activity of ohelo berry may be a result of a synergy between its organic acid and phenolic contents. 

Biofilm is a complex surface attachment community composed of various microorganisms, which are held together by extracellular polymer substances they produce [42]. About 60% of foodborne illness outbreaks and 80% of bacterial infections are related to biofilms [43]. *L*. *monocytogenes* biofilms formed in food processing environments are resistant to sanitizers and hard to eradicate, which poses a serious food safety concern. Dushku et al. [44] reported that the biofilm formation capability of *L*. *monocytogenes* was associated with cell surface properties, such as hydrophobicity and auto-aggregation, which were essential for its increased tolerance to gut barriers, survival in the host, and hypervirulence activity. Hydrophobicity influences bacterial adhesion, proliferation, and distribution. Fan et al. [45] reported that *L*. *monocytogenes* strains with higher hydrophobicity exhibited stronger adhesion and biofilm-forming capacity. Choi et al. [46] also showed that biofilm formation of *L. monocytogenes* was positively correlated with its hydrophobicity level. The present study demonstrated that sub-inhibitory concentrations of ohelo berry juice could significantly decrease the hydrophobicity and biofilm formation capability of *L. monocytogenes* in a concentration-dependent manner. Moreover, changes in bacterial hydrophobicity may alter surface tensions between bacterial cells and result in a tendency of bacterial cells to attach to each other and form aggregates [47,48]. Interestingly, the present study revealed that 6.25% ohelo berry juice indeed increased the auto-aggregation of *L*. *monocytogenes*, while the biofilm formation of *L*. *monocytogenes* was inhibited with reducing hydrophobicity (Table 3). In contrast, Kim et al. [49] found that the cell-free supernatant of probiotic *Saccharomyces cerevisiae* could inhibit the biofilm formation of *L. monocytogenes* along with decreasing auto-aggregation and hydrophobicity. Nevertheless, Choi et al. [46] reported that the hydrophobicity and auto-aggregation capability of *L. monocytogenes* might be two independent traits, though both were necessary for adhesion. Wang et al. [48] investigated the effects of extracts from cranberry on five *L. monocytogenes* strains isolated from raw chicken with respect to their hydrophobicity and auto-aggregation. They reported that significant increases of hydrophobicity and auto-aggregation of *L. monocytogenes* ATCC 7644 were observed in the presence of cranberry, though a significant increase of hydrophobicity along with a significant decrease of auto-aggregation was found for cranberry-treated *L. monocytogenes* B2. The results showed that the changes of hydrophobicity and auto-aggregation were dependent on the type of *L. monocytogenes* strains. 

Motility is another important property of bacteria that plays a crucial role in initial attachment and the spread of bacteria on a surface for subsequent biofilm formation [26,50]. The swimming motility refers to the flagella-directed movement in aqueous environments, whereas swarming motility is the flagella-directed rapid movement on solid surfaces [26]. Dons et al. [51] showed that the swarming motility of *L. monocytogenes* was critical in initial contact with epithelial cells and contributed to the effective invasion of host cells. Our study revealed that sub-inhibitory concentrations of ohelo berry juice could significantly decrease the swarming and swimming motility of *L. monocytogenes*, highlighting the promise of ohelo berry as a potential agent to suppress the movement of *L. monocytogenes* and sequentially reduce the infection. 

To further understand the antimicrobial property of ohelo berry juice, expression patterns of six genes in treated *L*. *monocytogenes* cells were examined. The target genes included *hly*, *iap*, *plc*A, *lmo*1666, *sig*B, and *fla*A (Table 1). The *fla*A gene and *sig*B gene play an important role in the motility and biofilm formation of cells [31]. Down-regulation of these two genes by 6.25% ohelo berry juice might inhibit the motility and biofilm formation of *L*. *monocytogenes* (Figure 3), in agreement with physicochemical property and biofilm formation tests (Table 3). However, the juice treatments did not significantly affect the expression of the *lmo*1666 gene (Figure 3), which encodes *Listeria* adhesion protein B [30]. Adhesion is an important step in biofilm formation [31]. Reduced biofilm formation of *L. monocytogenes* by sub-inhibitory concentrations of ohelo berry juice could be due to the down-regulation of other adhesion-related genes in *L*. *monocytogenes*, such as *lmo*1634 and *lmo*1847 [30]. Finally, the *iap* gene encodes a protein responsible for the invasion of host cells by *L*. *monocytogenes* [30]. The *hly* gene codes for listeriolysin O, which enables *L*. *monocytogenes* cells to escape phagosome and cause infection [52]. The *plc*A gene encodes phosphatidylinositol phospholipase C [53]. The down-regulated expression of these three genes suggested that ohelo berry juice could prevent *L. monotytogenes* infection by inhibiting the production of virulence factors by *L*. *monocytogenes* (Figure 3).

Milk was used as a model to examine the antimicrobial property of ohelo berry juice in food matrices. It is conceivable that the effectiveness of antimicrobials would be weakened in food than in broth media because of possible interactions of antimicrobials with food components, inactivation by enzymatic modification, poor solubility, and uneven distribution [2,54,55]. The present study showed that *L. monocytogenes* in milk increased in 72 h from 6 log to 7.3 and 8.5 log CFU/mL at 7 and 37 °C, respectively (Figure 4). Complete elimination of *L. monocytogenes* was not achieved with the tested concentrations of ohelo berry juice in our study, but cell counts of *L. monocytogenes* were significantly reduced by 1 to 2 log CFU/mL in milk supplemented with 50% ohelo berry juice at 37 °C for 72 h. Furthermore, the 50% juice exhibited bacteriostatic effects on *L. monocytogenes* in milk throughout incubation at 7 °C. Similar observations were reported by Biswas et al. [56], who investigated the effectiveness of blueberry juice mixed with skim milk (1:1) on the growth of *L. monocytogenes*. They found that the counts of *L. monocytogenes* were reduced by about 1.5 log CFU/mL after a 72-h incubation at 37 °C. Additionally, Yang et al. [55] concluded that the growth of *L. monocytogenes* was inhibited in both whole and skim milk supplemented with 10% blackberry juice stored at 37 °C for 72 h. No significant difference was observed between whole milk and skim milk. Similarly, 50% ohelo berry juice proved to be effective in reducing the number of *L. monocytogenes* in both whole and skim milk, revealing that the fat content of milk did not interfere with the interaction of bioactive compounds in ohelo berry juice with bacterial cells. In contrast, the antimicrobial properties of nisin and glycolipids against *L. monocytogenes* in milk were significantly reduced by its fat content [57,58].

## 5. Conclusions

This study showed the ohelo berry juice exerted effective antimicrobial activities against *L. monocytogenes*. Sublethal concentrations of ohelo berry juice significantly increased auto-aggregation and decreased hydrophobicity, swimming motility, swarming motility, and biofilm formation capability of *L. monocytogenes*, highlighting potential applications of ohelo berry juice to tackling hygienic and sanitary issues in the food industry. Furthermore, the expression of motility, biofilm formation, and virulence-related genes in *L. monocytogenes* was down-regulated by the juice treatments. *L. monocytogenes* was significantly inhibited in whole and skim milk supplemented with 50% ohelo berry juice regardless of fat content, underlining the potential of ohelo berry as a natural food preservative to control foodborne pathogens in milk products. This is the first report on the antimicrobial property of ohelo berry. It is worth further exploring the application of ohelo berry in broad food matrices, such as unpasteurized cheese, and its influence on the sensory and nutritional properties of ohelo berry-supplemented products. 

## Figures and Tables

**Table 1:** Information about the primers used in Quantitative PCR.

Gene	Primer	Sequence	Locus	Protein/Function	Reference	Efficiency ^1^
16S *rRNA*	Forward	5′-TGGCGGACGGGTGAGTA-3′	lmor01	Reference gene	Upadhyay et al. [30]	2.05
	Reverse	5′-CCGGAGTTATCCCCAACTTACA-3′				
*hly*	Forward	5′-TCTCCGCCTGCAAGTCCTA-3′	lmo0202	Listeriolysin O	Upadhyay et al. [30]	2.06
	Reverse	5′-TCGATTTCATCCGCGTGTT-3′				
*iap*	Forward	5′-CTACAGCTGGGATTGCGGTAA-3′	lmo0582	Invasion-associated protein	Upadhyay et al. [30]	1.99
	Reverse	5′-TGCTTGCGGATGCGATT-3′				
*lmo*1666	Forward	5′-TGGAGTGGGCACGTGTTGT-3′	lmo1666	Adhesion protein B	Upadhyay et al. [30]	2.03
	Reverse	5′-TTGTCAGCTGCATATTGTGAATTG-3′				
*plc*A	Forward	5′-TCGGACCATTGTAGTCATCTTGA-3′	lmo0201	Phospholipase C	Upadhyay et al. [30]	1.97
	Reverse	5′-CACAAATTCGGCATGCAGTT-3′				
*sig*B	Forward	5’-GATGATGGATTTGAACGTGTGAA-3’	lmo0895	Biofilm formation and disinfectant resistance	Li et al. [31]	1.99
	Reverse	5’-CGCTCATCTAAAACAGGAGAAC-3’				
*fla*A	Forward	5’-CTGGTATGAGTCGCCTTAG-3’	lmo0690	Flagellin	Li et al. [31]	1.96
	Reverse	5’-CATTTGCGGTGTTTGGTTTG-3’				

^1^ A standard curve was generated from the 10-fold serially diluted complementary DNA (cDNA) derived from RNA extract from *L. monocytogenes* cells, with an identified threshold cycle number (Ct) and slope of the trend line. The efficiency was calculated using the equation: E = −1 + 10^(−1/slope) [28].

**Table 2:** The pH values of diluted ohelo berry juice.

Concentration	pH
100%	3.27 ± 0.03 a ^1^
50%	3.86 ± 0.04 b
25%	4.24 ± 0.02 c
12.5%	4.69 ± 0.04 d
6.25%	5.40 ± 0.01 e
3.12%	6.37 ± 0.02 f
1.56%	6.85 ± 0.05 g
0.78%	7.09 ± 0.01 h

^1^ Means with different letters (a through h) are significantly different at *p* < 0.05.

**Table 3:** Physicochemical properties and biofilm formation capability of *L. monocytogenes* in the presence of ohelo berry juice ^1^.

Ohelo Berry Juice (%)	Hydrophobicity (%)	Auto-Aggregation (%)	Swimming Motility ^2^ (%)	Swarming Motility ^2^ (%)	Biofilm Formation ^2^ (%)
0	7.71 ± 0.85 a	16.02 ± 2.83 a	100 a	100 a	100 a
3.12	2.77 ± 0.46 b	23.95 ± 3.83 a	41.29 ± 3.00 b	22.80 ± 0.31 b	54.33 ± 0.39 b
6.25	2.14 ± 0.87 b	67.98 ± 10.92 b	4.39 ± 0.17 c	14.71 ± 0.23 b	23.00 ± 0.53 c

^1^ Means with different letters in the same column (a through c) are significantly different at *p* < 0.05. ^2^ Motility and biofilm formation values were normalized to untreated control.

## Data Availability

Not applicable.
